# Impact of Summer Heat on Urban Population Mortality in Europe during the 1990s: An Evaluation of Years of Life Lost Adjusted for Harvesting

**DOI:** 10.1371/journal.pone.0069638

**Published:** 2013-07-22

**Authors:** Michela Baccini, Tom Kosatsky, Annibale Biggeri

**Affiliations:** 1 Department of Statistics, Informatics and Applications “G. Parenti”, University of Florence, Florence, Italy; 2 Biostatistics Unit, ISPO Cancer Prevention and Research Institute, Florence, Italy; 3 WHO European Centre for Environment and Health, Rome, Italy; Iran University of Medical Sciences, Islamic Republic of Iran

## Abstract

**Background:**

Efforts to prevent and respond to heat-related illness would benefit by quantifying the impact of summer heat on acute population mortality. We estimated years of life lost due to heat in 14 European cities during the 1990s accounting for harvesting.

**Methods:**

We combined the number of deaths attributable to heat estimated by the PHEWE project with life expectancy derived from population life tables. The degree of harvesting was quantified by comparing the cumulative effect of heat up to lagged day 30 with the immediate effect of heat, by geographical region and age. Next, an evaluation of years of life lost adjusted for harvesting was obtained.

**Results:**

Without accounting for harvesting, we estimated more than 23,000 years of life lost per year, 55% of which was among individuals younger than 75. When 30 day mortality displacement was taken into account, the overall impact reduced on average by 75%. Harvesting was more pronounced in North-continental cities than in Mediterranean cities and was stronger among young people than among elderly.

**Conclusions:**

High ambient temperatures during summer were responsible for many deaths in European cities during the 1990s, but a large percentage of these deaths likely involved frail persons whose demise was only briefly hastened by heat exposure. Differences in harvesting across regions and classes of age could reflect different proportions of frail individuals in the population or could be indicative of heterogeneous dynamics underlying the entry and exit of individuals from the high-risk pool which is subject to mortality displacement.

## Introduction

Quantifying the impact of heat on acute population mortality strengthens public health authorities in advocating for a coordinated heat response and in arguing for heat-resilient adaptations to housing and urban design. Relative risk measures are not adequate for this purpose, as they do not take into account the absolute likelihood of disease or death [1]. For example, small relative risks translate to strong impacts at the population level if the fraction of the exposed population is large, while large relative risks produce negligible impacts if the exposure is rare. Methods for health impact assessment (HIA), which combine relative risk with prevalence and level of exposure, are needed.

The effect of heat on population mortality is usually quantified in terms of an absolute number of deaths or of a fraction of deaths attributable to the exposure. The calculation of years of life lost (YLL) represents an extension of attributable deaths and attributable fraction [2]. This indicator accounts for life expectancy in the absence of the exposure of interest, providing an estimate of premature mortality related to heat. In quantifying years of life lost due to heat, the problem arises of whether the life expectancy of individuals who die during periods of hot temperature is comparable to or shorter than the life expectancy of the general population. This is a crucial issue which is directly related to the public health importance of heat-related mortality [3].

It is often asserted that heat-related deaths largely involve frail persons whose impending demise is hastened by heat exposure. This phenomenon has been termed “mortality displacement” or “harvesting”, and it is reflected by an immediate excess in population mortality following a temperature rise, with a subsequent compensatory reduction of mortality. Distributed lag models on time series data have often been used to study this phenomenon. Braga *et al.* (2001) found that the apparent hot temperature effect in major US cities was primarily due to mortality displacement [4]. Hajat *et al.*(2005) compared the extent to which short-term mortality displacement could explain excess hot day deaths in Delhi, São Paulo and London [5]; they found that mortality displacement was high in London where the excess of heat-related deaths persisted for only 2 days after exposure and was followed by a deficit, suggesting that the pool of susceptible individuals was exhausted within days of the temperature peak. Recently, Guo *et al*. (2011) measured heat harvesting in Tianjin, China and found that heat-related excesses of deaths due to cardiopulmonary and cardiovascular causes were followed by deficits in mortality during the next 5–20 days [6]. Rocklov and Forsberg (2010) showed mortality displacement in elderly persons in Sweden, and Klenk *et al.* (2010) reported that the excess of deaths after 3 months among elderly nursing home residents still constituted more than 80% of the excess observed after the first month [7], [8].

On the other hand, some studies have shown little evidence of harvesting. Basu and Malig (2011) concluded that there was no heat-related harvesting in California counties and Toulemon and Barbieri (2008), focusing on the 2003 heat wave in France, found that the level of mortality during the 10 days immediately following did not show a dip [9], [10]. However it should be noticed that heat waves and regular high temperatures may not have the same pattern of mortality displacement [11].

The aim of this paper is to estimate the loss of life expectancy of European urban populations through their exposure to summertime heat. Baccini et al. (2011) evaluated the number of deaths attributable to high ambient temperatures in 15 European cities during the 1990s, using mortality counts, temperature distributions and relative risks generated through the PHEWE study [12], [13]. In this research it was found that high summer temperatures have an important impact on European population health and that heat-attributable deaths can be expected to increase markedly under warming scenarios. Here we extended this evaluation, estimating for the same cities YLL related to the impact of heat. We addressed harvesting and proposed a simple evaluation of YLL to account for it.

## Data and Methods

We considered the effect of daily maximum apparent temperature on mortality for all natural causes (International Classification of Diseases-9 codes 1–799) during the warm season in the cities enrolled in the PHEWE project: Athens, Barcelona, Budapest, Dublin, Helsinki, Ljubljana, London, Milan, Paris, Prague, Rome, Stockholm, Turin, Valencia, Zurich [14]. The study period was not identical for all cities, but consisted of at least 5 consecutive years between 1990 to 2000. The warm season was defined as the period from April 1 to September 30. Exposure was measured by daily maximum apparent temperature, calculated from 3-hourly air temperature and humidity data. For Barcelona, the daily average apparent temperature was used, as 3-hourly data was not available.

In Baccini et al. (2011), the PHEWE results were used to calculate the number of deaths attributable to maximum apparent temperatures exceeding the city-specific threshold [12]. Attributable mortality was calculated assuming that increases of daily maximum apparent temperature under a city-specific threshold did not affect mortality and that the heat effect was linear (on a log scale) above this threshold. In the present paper, we extended this evaluation calculating loss of life expectancy associated with maximum apparent temperatures above the threshold.

For calculating YLL attributable to heat exposure, we related the number of attributable deaths for each age class to the average life expectancy in that class, obtained from population life tables. Life tables for a reference year between 1993 and 2000 (by single year of age or five-year classes) were acquired or developed by city. A life table was not available for Prague, which was excluded from YLL calculation. We assumed that the age distribution of the excess of deaths by day within each large class of age (15–64, 65–74, 75+) was equal to that of all deaths occurring during the reference year. We calculated YLL by multiplying the number of attributable deaths in the *h*th class of age (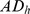) by the average life expectancy within that class (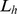):



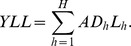
.

This YLL estimate is based on the assumption that the life expectancy of the individuals who die due to heat is the same as that of the general population. This assumes that there is no harvesting.

### Harvesting Effect

To address harvesting, we explored the lagged effect of heat by geographical region and age class. First, for each city (including Prague for consistency with the PHEWE study) and age class, we investigated the lagged effect of daily maximum apparent temperatures above the city-specific threshold up to 30 days using unrestricted distributed lag models, which provided an unbiased estimate of the final cumulative effect of the exposure [15]. Second, we combined these city-specific results, separately by geographical region, in a Bayesian meta-analysis, obtaining a posterior estimate of the overall cumulative effect for each age class [16]. According to the PHEWE protocol, we considered the following geographical regions: the Mediterranean region (including Athens, Rome, Barcelona, Valencia, Turin, Milan and Ljubljana) and the North-continental region (including Budapest, Zurich, Paris, Prague, Helsinki, Stockholm, London and Dublin). The age-specific degree of harvesting was quantified by one minus the proportion *k* of the lag 30 over the lag 3 overall cumulative effect, the lag 3 overall cumulative effect being that reported in Baccini et al. (2008) [13]. In the presence of harvesting, *k* is expected to be lower than one, being the cumulative effect up to 30 days lower than the cumulative effect up to 3 days. Values of *k* close to one indicate absence of harvesting. Finally, we estimated the years of life lost net of harvesting, by multiplying the proportion *k* by the years of life lost obtained for each city before adjusting for mortality displacement. This is an approximate calculation, but it should provide reliable results under specific conditions on the apparent temperature and mortality time series, which resulted to be roughly satisfied in the present data set (see Appendix S1 for more detail).

It should be noticed that the value of *k* depends on the choice of the time window over which one evaluates the lagged effect of exposure. On the one hand, if the time window is not wide enough to include possible mortality rebound, *k* will be overestimated. On the other, defining an excessively wide time window can likely lead to unstable results. We selected a 30 day window on the basis of previous PHEWE results, which indicated a strong positive effect of heat within the first week, which declined in subsequent days, became negative, then returned to the level of baseline mortality after one month in the Mediterranean cities and after 20 days in the Northern cities [13].

To have an idea of the uncertainty around the degree of harvesting, we used a Monte Carlo approach. We assumed a multivariate normal distribution on lag 3 and lag 30 cumulative percent changes, we sampled 1,000 pairs of values from this distribution and calculated the quantity 1−*k* from each pair, obtaining a sample from the distribution of the degree of harvesting. We evaluated the variability of the degree of harvesting by looking at the percentiles of this sample.

## Results

In Table 1, we present demographic and climatic descriptors of the 15 cities enrolled in the PHEWE study. A large variability in maximum apparent temperatures was observed among cities. The impact of summertime heat ranges from approximately 0 attributable deaths per year in Dublin to 423 in Paris [12]. The total number of attributable deaths in the 15 cities was around 2,300 per year (2234 if we exclude Prague).

**Table 1:** Study period, population size, percentage of elderly, mean of daily maximum apparent temperature during the warm season, deaths attributable to heat per year, by city.

City	Study period	Population	% over 75	Max AT (°C)	AD per year**
**Athens**	1992–1996	3188305	6.4	27.6	230
**Barcelona**	1992–2000	1512971	10.1	23.3*	290
**Budapest**	1992–2001	1797222	7.3	21.8	399
**Dublin**	1990–2000	481854	5.3	14.7	0
**Helsinki**	1990–2000	955143	5	14.3	11
**Ljubljana**	1992–1999	263290	5.9	20.1	13
**London**	1992–2000	6796900	6.8	18	142
**Milan**	1990–2000	1304942	9.5	25.4	95
**Paris**	1991–1998	6161393	6.1	19.5	423
**Prague**	1992–2000	1183900	7	17.8	72
**Rome**	1992–2000	2812573	7.3	26	388
**Stockholm**	1900–2000	1173183	8.5	15.3	21
**Turin**	1991–1999	901010	9.2	23.4	121
**Valencia**	1995–2000	739004	7.2	29.5	72
**Zurich**	1990–1996	990000	7.8	19	29

Note: % over 75 = percentage of the population over 75 years of age; Max AT = mean of daily maximum apparent temperature during the warm season (April to September); AD per year = deaths attributable to heat per year (rounded values).

* Mean of daily mean apparent temperature.

** Results from Baccini et al. (2011).

Average life expectancy ranges from 24.1 years (Budapest) to 34.2 years (Paris) for individuals belonging to the first class of age, from 11.3 years (Budapest) to 17.7 years (Paris) for the second, and from 4.7 (Ljubljana) to 8.8 years (London) for the oldest (Table 2).

**Table 2:** Average life expectancy by age class.

		Life expectancy		
		Age class		
City	Reference year	15–64	65–74	75+
**Athens**	1991	30.0	14.8	7.8
**Barcelona**	1995	31.4	14.4	5.8
**Budapest**	1996	24.1	11.3	5.1
**Dublin**	1996	29.9	13.9	7.1
**Helsinki**	1996	29.2	13.0	5.2
**Ljubljana**	1995	25.6	12.2	4.7
**London**	1998	31.8	16.2	8.8
**Milan**	1998	30.2	15.2	6.7
**Paris**	1998	34.2	17.7	7.7
**Rome**	1998	29.2	13.9	6.5
**Stockholm**	2000	32.0	16.4	7.5
**Turin**	1998	29.4	14.5	6.7
**Valencia**	1996	30.0	13.2	5.5
**Zurich**	1995	31.3	14.0	5.9

In Table 3, we report the total and age-class specific expected YLL per year during the study period with the associated 80% credibility interval, by city, before accounting for harvesting. The lower and upper bounds of the 80% credibility interval are defined as the 10th and the 90th percentiles of the posterior distribution of the YLL per year. On aggregate, maximum apparent temperature above the city-specific threshold was responsible for 23,750 years of life lost per study year, 55% of which was among individuals younger than 75 (7,899 years of life lost in the first class of age, 5,067 in the second and 10,784 in the elderly). The largest annual average impact in terms of absolute number of YLL was observed in Budapest (3,891), Paris (5,483) and Rome (3,940).

**Table 3:** Years of life lost attributable to heat per year before accounting for harvesting.

	Age class							
	15–64		65–74		75+		Total (15+)	
City	YLL	80% CrI	YLL	80% CrI	YLL	80% CrI	YLL	80% CrI
**Athens**	287	(34,645)	628	(376,897)	1385	(1031,1729)	2300	(1441,3272)
**Barcelona**	617	(109,1195)	440	(101,792)	1401	(1024,1807)	2458	(1233,3793)
**Budapest**	1710	(1204,2205)	918	(703,1141)	1263	(1074,1475)	3891	(2980,4822)
**Dublin**	3	(0,7)	2	(0,4)	2	(0,4)	7	(1,15)
**Helsinki**	95	(33,157)	43	(18,72)	24	(7,41)	162	(58,270)
**Ljubljana**	58	(3,157)	25	(1,65)	43	(5,104)	126	(9,327)
**London**	674	(305,1076)	382	(157,617)	858	(571,1149)	1914	(1033,2841)
**Milan**	145	(29,294)	251	(149,362)	497	(361,646)	893	(539,1302)
**Paris**	2316	(1644,3025)	744	(465,1029)	2423	(2047,2816)	5483	(4156,6869)
**Rome**	1168	(812,1532)	947	(762,1131)	1825	(1589,2079)	3940	(3162,4742)
**Stockholm**	135	(16,284)	44	(11,80)	106	(62,153)	285	(89,516)
**Turin**	167	(27,337)	346	(187,525)	614	(408,852)	1127	(622,1714)
**Valencia**	407	(58,832)	185	(26,398)	244	(55,480)	836	(138,1710)
**Zurich**	117	(24,226)	112	(53,175)	99	(47,154)	328	(123,555)

Note: YLL = years of life lost per year before accounting for harvesting; 80% CrI = 80% credibility interval for YLL.

**Figure 1:**
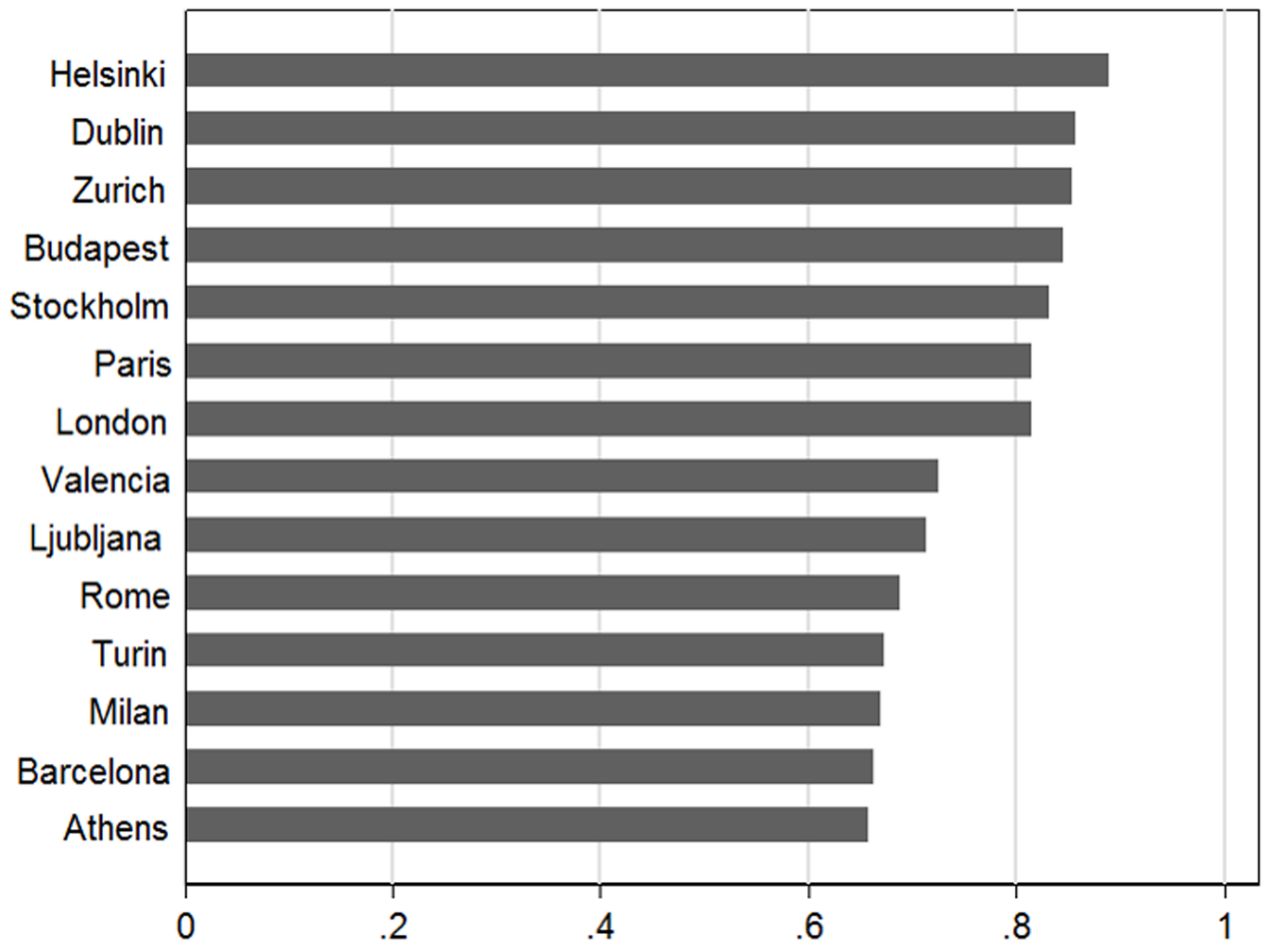
Proportional reduction in years of life lost when accounting for harvesting.

Table 4 presents posterior means and 90% credibility intervals of lag 3 and lag 30 cumulative percent change in total mortality associated with a 1°C increase in maximum apparent temperature above the threshold, by region and age class. These estimates come from the Bayesian meta-analyses of the city-specific distributed lag results; the lower and upper bounds of the 90% credibility interval are defined as the 5th and the 95th percentiles of the posterior distribution of the cumulative percent change. The percentage of mortality displacement was larger in the younger classes of age and in the North-continental cities. When we applied these percentages to the evaluated YLL, we found that maximum apparent temperature above the city-specific threshold accounted for 5,907 years of life lost per year in total: 1,051 in the first class of age, 685 in the second and 4,171 in the third (Table 5). In Helsinki, Dublin, Budapest, Stockholm, Paris and London the net (0–30 day lag) mortality burden was less than 20% of the burden obtained before accounting for harvesting. A smaller effect of harvesting was found in Athens, Barcelona, Milan, Turin and Rome. In these cities the net burden was more than 30% of that obtained before accounting for harvesting (Figure 1).

**Table 4:** Cumulative effect of heat on mortality up to lag 3 and up to lag 30 and harvesting quantification.

		Cumulative effect				
		up to lag 3**		up to lag 30		
Region	Age class	% change	(90% CrI)	% change	(90% CrI)	% harvesting
**Mediterranean** *	**15–64**	0.92	(−0.93, 2.81)	0.20	(−1.58, 2.00)	78.2
	**65–74**	2.13	(−0.01, 4.31)	0.41	(−1.31, 2.16)	80.6
	**75+**	4.22	(1.80, 6.70)	1.83	(0.17, 3.52)	56.1
**North-continental** *	**15−64**	1.31	(−0.57, 3.23)	0.11	(−1.45, 1.64)	91.5
	**65−74**	1.65	(−0.16, 3.49)	0.10	(−1.46, 1.67)	93.9
	**75+**	2.07	(0.54, 3.63)	0.66	(−0.86, 2.16)	67.9

Note: % change = posterior mean of cumulative percent change in total mortality associated with a 1°C increase of maximum apparent temperature above the city-specific threshold; 90% CrI = 90% credibility interval of cumulative percent change; % harvesting = percentage of harvesting quantified by 100×(1**–** *k*),where *k* is the ratio between lag 30 and lag 3 overall cumulative effect.

* Mediterranean cities: Athens, Rome, Barcelona, Valencia, Turin, Milan and Ljubljana.

North-continental cities: Budapest, Zurich, Paris, Prague, Helsinki, Stockholm, London and Dublin.

** Results from Baccini et al. (2008).

**Table 5:** Years of life lost attributable to heat per year adjusted for harvesting.

	Age class							
	15–64		65–74		75+		Total (15+)	
City	YLL	80% CrI	YLL	80% CrI	YLL	80% CrI	YLL	80% CrI
**Athens**	63	(7,141)	122	(73,174)	608	(453,759)	792	(533,1074)
**Barcelona**	135	(24,261)	85	(20,154)	615	(450,793)	835	(493,1207)
**Budapest**	145	(102,187)	56	(43,70)	405	(345,473)	607	(490,731)
**Dublin**	0	(0,1)	0	(0,0)	1	(0,1)	1	(0,2)
**Helsinki**	8	(3,13)	3	(1,4)	8	(2,13)	18	(6,31)
**Ljubljana**	13	(1,34)	5	(0,13)	19	(2,46)	36	(3,92)
**London**	57	(26,91)	23	(10,38)	275	(183,369)	356	(219,498)
**Milan**	32	(6,64)	49	(29,70)	218	(158,284)	298	(194,418)
**Paris**	197	(140,257)	45	(28,63)	778	(657,904)	1020	(825,1224)
**Rome**	255	(177,334)	184	(148,219)	801	(698,913)	1240	(1022,1466)
**Stockholm**	11	(1,24)	3	(1,5)	34	(20,49)	48	(22,78)
**Turin**	36	(6,73)	67	(36,102)	270	(179,374)	373	(221,549)
**Valencia**	89	(13,181)	36	(5,77)	107	(24,211)	232	(42,469)
**Zurich**	10	(2,19)	7	(3,11)	32	(15,49)	49	(21,80)

Note: YLL = years of life lost per year adjusted for harvesting; 80% CrI = 80% credibility interval for YLL.

The large credibility intervals we obtained for the cumulative effect estimates in the younger classes of age (Table 4) impact estimates of the percentage of harvesting and of the estimation of the YLLs. For the 75+ class of age, where the cumulative effect estimates were more stable, we tried to evaluate uncertainty around the percentage of harvesting via a Monte Carlo approach. Assuming a multivariate normal distribution on lag 3 and lag 30 cumulative percent changes, with correlation equal to 0.8, we obtained a large sample of values for the percentage of mortality displacement. We found a 50% Credibility Interval for the percentage of harvesting ranging from 46.1 to 66.8 in the Mediterranean region and from 46.4 to 95.1 in the North-continental region. These results appeared robust to different specification of the correlation level between the two cumulative percent changes.

A relative evaluation of the mortality burden was obtained in terms of age-specific YLL per year per 10000 inhabitants (Table 6). With reference to the net of harvesting estimates, the largest life expectancy reduction attributable to heat was observed in the elderly, with more than 30 years of life lost per year per 10,000 inhabitants in four Mediterranean cities (Athens, Barcelona, Rome, Turin) and in Budapest. Among the North-continental cities, Paris also stands out, with 21 years of life lost per 10000 persons over 75 years of age. Regarding the first two classes of age, before accounting for harvesting, the largest relative impact was observed in Budapest (13 YLL per year per 10000 inhabitants in the 15–64 class and 48 YLL per year per 10000 inhabitants in the 65–74 class); when accounting for harvesting, the larger relative impact was observed in the Mediterranean cities.

**Table 6:** Age-specific years of life lost attributable to heat per year per 10,000 population before accounting for harvesting, and adjusted for harvesting.

	YLL per year per 10,000 population					
	before accountingfor harvesting			adjusted for harvesting		
	Age class			Age class		
City	15–64	65–74	75+	15–64	65–74	75+
**Athens**	1.35	26.97	84.90	0.29	5.23	37.27
**Barcelona**	5.96	24.31	107.42	1.30	4.72	47.16
**Budapest**	13.36	48.33	97.40	1.14	2.95	31.27
**Dublin**	0.10	0.47	0.72	0.01	0.03	0.23
**Helsinki**	1.48	7.41	5.35	0.13	0.45	1.72
**Ljubljana**	3.08	10.67	34.23	0.67	2.07	15.03
**London**	1.39	7.95	19.40	0.12	0.48	6.23
**Milan**	1.63	16.00	41.33	0.36	3.10	18.14
**Paris**	5.48	17.06	65.82	0.47	1.04	21.13
**Rome**	6.35	34.39	97.00	1.38	6.67	42.58
**Stockholm**	1.71	5.41	10.60	0.15	0.33	3.40
**Turin**	2.67	31.91	78.28	0.58	6.19	34.36
**Valencia**	7.80	25.83	50.48	1.70	5.01	22.16
**Zurich**	1.45	11.81	12.70	0.12	0.76	4.08

## Discussion

This is the first study which evaluated the impact of high ambient temperatures on human health in terms of loss of life expectancy. We considered the period before the 2003 heat waves, which brought the risks of heat exposure to the attention of the media and the general public, encouraging the adoption of mitigation measures and public health interventions. During the 1990s, summer heat was responsible for more than 2,200 deaths per year in the 14 European cities considered in the analysis [12]. We estimated that, if the persons who die due to heat were not more frail than the general population, the years of life lost attributable to heat would have exceeded 23,000 per year in the 14 European cities, of which 55% were accounted for by the deaths of persons younger than 75. Deriving life expectancy from general population life tables however leads to overestimating YLL if the life expectancy of individuals who die due to heat is lower than that of the general population.

As in previous studies, our analysis indicated that many of the attributable deaths are one-month-displaced deaths, suggesting that the population of decedents is characterized by the presence of subgroups of very susceptible individuals for whom heat precipitates death by a few days up to few weeks [4]–[6]. We found that when these one-month-displaced deaths are removed, the overall impact in terms of YLL reduces by 75% for the cities assessed. The percentages of harvesting we found are consistent with Guo et al. (2011) who report for Tianjin a 2.03% increase in mortality associated with a 1°C increase of temperature above a 24.9°C threshold at lag0–2 and a 0.31% increase in mortality at lag0–27 [6].

The idea underlying harvesting is that, under steady-state conditions, there is equilibrium between the number of deaths each day and the daily net recruitment into the high-risk pool. If the exposure to the transient environmental factor (i.e. heat) increases mortality among these frail individuals, but does not increase their recruitment, the high-risk pool becomes temporary smaller, with fewer people who die [17]. The time needed to replenish the high-risk pool is related to the life expectancy of the frail individuals [18]. Our procedure attributed zero life expectancy to one-month displaced deaths; a more conservative approach would attribute one month of life lost to these deaths. In theory, we would not observe harvesting when the life expectancy of the people who die due to heat is longer than a few weeks or when heat affects frail individuals by briefly hastening their death, but at the same time increases recruitment into the high-risk pool. Given this last consideration, it is possible that we underestimated the real effect of harvesting, with an overestimation of YLL attributable to heat.

We found that harvesting is more pronounced in the North-continental than in the Mediterranean cities. This result may indicate that the percentage of deaths which are only briefly displaced by heat, over the total number of deaths attributable to heat, is larger in the former. This difference could be a reflection of varying proportions of frail individuals in the two regions, as related to specific socio-economic and demographic factors. An alternative explanation could be that in the North-continental region, where the level of exposure is lower, high temperatures hasten deaths of individuals belonging to the high-risk pool, but do not cause the recruitment of new individuals in the pool, so that high harvesting is observed. On the contrary, the exit/entry flows from/to the high-risk pool are more balanced in the Mediterranean region, so that harvesting appears lower. In other words, in the Mediterranean cities heat could not only act as a precipitating factor for mortality in high-risk individuals, but could also induce morbidity or exacerbate pre-existing non-severe diseases, so that new individuals become part of the high-risk pool. Similar reasoning could explain why we observed higher levels of harvesting among the younger population than among the elderly: in the younger classes of age, heat could precipitate deaths of high-risk individuals, but not increase at the same rate the recruitment of new individuals into the high-risk pool, because of the general robustness of this segment of the population.

However, in interpreting differences across regions and classes of age, one should consider that our harvesting evaluation could be sensitive to the threshold definition and to the choice of the model for the exposure-response relationship. In the North-continental region the threshold was around 23.3°C, so that the linear term captured the effect of relatively low apparent temperatures. These temperatures were not considered in the health impact evaluation for the Mediterranean region, where attributable deaths were referenced to apparent temperatures above a 29.4°C threshold. This consideration, coupled with the assumption that frail persons may be susceptible to apparent temperatures much lower than individual average, indicates that we could have underestimated harvesting in the Mediterranean region. Similarly, the same threshold was used for the three classes of age, because estimating age-specific thresholds provided very unstable results [13], [19]. With this choice we could have underestimated the harvesting in the over 65 class, where a lower apparent temperature threshold could in principle be more appropriate. Methods which simultaneously model mortality displacement and shape of the exposure-response curve could be used to better explore these points.

Finally, we note that our study did not consider long term mortality displacement. In fact, even when heat does not precipitate imminent death, the individuals who die due to heat could have a shorter life expectancy than the general population, because underlying medical conditions may make individuals more susceptible to hot weather, or, simply, because their age distribution within the broad classes defined for the analysis could be different from that present in the general population [20]–[23]. In our evaluation we did not take this into account and estimated YLL using life expectancy from current general population life tables. Alternative life expectancy assumptions, for example considering life expectancy among persons with pre-existing cardiac or respiratory disease, would have led to lower estimates of YLL.

In conclusion, our study indicates that while high ambient temperatures during summer have been responsible for many deaths in European cities in the 1990s, a large percentage involved frail persons whose life expectancy ranged from one week to one month. In these cases heat possibly acted as a precipitating factor for near-term mortality. As a consequence, loss of life expectancy was lower than that expected in the absence of harvesting. This suggests that the impact of heat in terms of YLL is strongly related to the presence of highly susceptible individuals in the population. We observed some differences in harvesting across regions and classes of age. These differences may reflect the presence of different proportions of frail individuals or could be indicative of heterogeneous dynamics influencing the entry and exit of individuals from the high-risk pool which is subject to mortality displacement.

## Supporting Information

Appendix S1 **Approximated calculation of attributable deaths net of harvesting.**
(DOCX)
