# Supplementary material for: Impact of Summer Heat on Urban Population Mortality in Europe during the 1990s: An Evaluation of Years of Life Lost Adjusted for Harvesting
Source: PLoS One. 2013 Jul 22;8(7):e69638. doi: 10.1371/journal.pone.0069638 (PMC3718689; doi:10.1371/journal.pone.0069638)
Supplement: Appendix S1 — Approximated calculation of attributable deaths net of harvesting. (DOCX) [file pone.0069638.s001.docx]

**Appendix S1**

For each city and class of age, let $\left(\beta_{0},\beta_{1},\ldots,\beta_{30}\right)$ be the estimated coefficients from the unrestricted distributed lag model describing the effect of the exposure from lag 0 to 30, and$\beta$ be the estimate of the slope above the threshold for the average maximum apparent temperature of the current and the previous three days arising from the PHEWE study (13). While $\beta$ roughly captures the cumulative effect of heat up to 3 days, $\sum_{j=0}^{30} \beta_{j}$ captures the cumulative effect of heat up to 30 days.

Separately by geographical region and class of age, we calculated the number of attributable deaths which are not one-month-displaced (*AD**) as the product between the total number of attributable deaths (*AD*) and the ratio (*k*) between the meta-analytic estimate of the cumulative effect up to 30 days and the meta-analytic estimate of the cumulative effect up to 3 days:

$AD^{*}=kAD$ . (1)

As a consequence, the years of life lost after the removal of harvesting (** ) were obtained as the fraction of the years of life lost calculated without accounting for mortality displacement:

$YLL^{*}=kYLL$ . (2)

The equations (1) and (2) provide approximated results and rely on the fact that, under specific conditions on the exposure and mortality time series$AD^{*}\approx kAD$, where$k=\sum_{j=0}^{30} \beta_{j}/\beta$. Given a specific city, let us assume that $x_{i}$ and $y_{i}$ are the maximum apparent temperature and the number of deaths in the *i*th day, respectively; $\bar{x}_{i}^{\left(0-3\right)}$ is the average maximum apparent temperature over the day *i* and the previous 3 days and *T* is the threshold above which we count the attributable deaths.

It is possible to shows the following relations:

$$\begin{matrix} AD=\sum_{i} \left(y_{i}-\frac{y_{i}}{\exp\left(\beta\left(\bar{x}_{i}^{\left(0-3\right)}-T\right)\mathrm{I}\left(\bar{x}_{i}^{\left(0-3\right)}>T\right)\right)}\right)\approx^{\left(a\right)} \sum_{i} \left(y_{i}-y_{i}\left(1-\beta\left(\bar{x}_{i}^{\left(0-3\right)}-T\right)\mathrm{I}\left(\bar{x}_{i}^{\left(0-3\right)}>T\right)\right)\right)= \\ =\beta\sum_{i} y_{i}\left(\bar{x}_{i}^{\left(0-3\right)}-T\right)\mathrm{I}\left(\bar{x}_{i}^{\left(0-3\right)}>T\right) \end{matrix}$$

$$\begin{matrix} AD^{*}=\sum_{i} \left(y_{i}-\frac{y_{i}}{\exp\left(\sum_{j=0}^{30} \beta_{j}\left(x_{i-j}-T\right)\mathrm{I}\left(x_{i-j}>T\right)\right)}\right)\approx^{\left(a\right)} \sum_{i} \left(y_{i}-y_{i}\left(1-\sum_{j=0}^{30} \beta_{j}\left(x_{i-j}-T\right)\mathrm{I}\left(x_{i-j}>T\right)\right)\right)= \\ =\sum_{i} y_{i}\sum_{j=0}^{30} \beta_{j}\left(x_{i-j}-T\right)\mathrm{I}\left(x_{i-j}>T\right)\approx^{\left(b\right)} \frac{1}{31}\sum_{i} y_{i}\sum_{j=0}^{30} \left(x_{i-j}-T\right)\mathrm{I}\left(x_{i-j}>T\right)\sum_{j=0}^{30} \beta_{j}= \\ =\frac{1}{31}\sum_{j=0}^{30} \beta_{j}\sum_{i} y_{i}\sum_{j=0}^{30} \left(x_{i-j}-T\right)\mathrm{I}\left(x_{i-j}>T\right)= \\ =k\beta\sum_{i} \frac{1}{31}y_{i}\sum_{j=0}^{30} \left(x_{i-j}-T\right)\mathrm{I}\left(x_{i-j}>T\right)\approx^{\left(c\right)} k\beta\sum_{i} y_{i}\left(\bar{x}_{i}^{\left(0-3\right)}-T\right)\mathrm{I}\left(\bar{x}_{i}^{\left(0-3\right)}>T\right)=kAD \end{matrix}$$

(a) Taylor’s approximation.

(b) if the correlation between $\left(x_{i-j}-T\right)\mathrm{I}\left(x_{i-j}>T\right)$ and $\beta_{j}$ *j*=0,1,…30 is close to zero for each *i*.

(c) if the correlation between$\sum_{j=0}^{30} \left(x_{i-j}-T\right)\mathrm{I}\left(x_{i-j}>T\right)$ and $y_{i}$ is close to the correlation between$\left(\bar{x}_{i}^{\left(0-3\right)}-T\right)I\left(\bar{x}_{i}^{\left(0-3\right)}>T\right)$ and$y_{i}$, and the mean of $\frac{1}{31}\sum_{j=0}^{30} \left(x_{i-j}-T\right)\mathrm{I}\left(x_{i-j}>T\right)$ and the mean of $\left(\bar{x}_{i}^{\left(0-3\right)}-T\right)\mathrm{I}\left(\bar{x}_{i}^{\left(0-3\right)}>T\right)$ over the study period are similar.

The assumptions (b) and (c) have been checked on the data sets used for the analysis and we found that they are roughly satisfied.
